# Straw Mulching Differentially Shapes the Structure and Function of Below‐Ground Bacterial Communities in Potato Depending on eDNA Source and Cultivar

**DOI:** 10.1002/pei3.70131

**Published:** 2026-02-19

**Authors:** Lovely Mahawar, Arti Mishra, Angeliki Tsitouri, Benedicte Riber Albrectsen

**Affiliations:** ^1^ Department of Plant Physiology Umeå University, Umeå Plant Science Centre Umeå Sweden; ^2^ Department of Botany Hansraj College, University of Delhi Delhi India

**Keywords:** bacterial communities, cv *King Edward*, cv *Mandel*, illumina amplicon sequencing, metabarcoding

## Abstract

Potato is the world's third most important food crop, yet its production relies heavily on pesticides, creating a need for sustainable alternatives. We assessed how straw mulching, a practice known to improve soil fertility, enrich microbial activity, and suppress diseases, affects below‐ground bacterial community structure and functional potential across different potato‐associated sample types. A field experiment was conducted in northern Sweden using two potato cultivars under mulched and control soil conditions. Samples from the rhizosphere, root, soil, and tuber peel were analyzed using 16S ribosomal RNA (rRNA) gene sequencing (Illumina platform) to assess bacterial diversity and community composition. Straw mulching significantly increased bacterial richness and altered community structure across sample types and cultivars. Copiotrophic genera, which thrive in nutrient‐rich environments, included *Rhodanobacter*, *Mucilaginibacter*, *Flavobacterium*, and *Pseudomonas*, and were enriched in rhizosphere, root, and tuber peel. Oligotrophs such as *Bryobacter* and *Candidatus Solibacter* dominated the soil and are known to contribute to organic matter turnover and plant growth. Notably, in the peel of one cultivar (*King Edward*), the abundance of *Pseudomonas* increased 5–7‐fold, correlating with elevated starch and ascorbic acid contents of the tubers. In conclusion, the effect of straw mulching on soil bacterial communities and tuber quality appears to be diverse and cultivar dependent. Long‐term and large‐scale studies are needed to evaluate cumulative impacts on soil health, yield, and resilience.

## 1 Introduction

Feeding a growing global population under accelerating climate change and emerging biological threats is one of the greatest challenges facing modern agriculture. By 2050, global food demand is projected to increase by 70%, driven by population growth, shifting climate patterns, declining efficiency of agrochemicals, and geopolitical instabilities (FAO 2009). Potato ( *Solanum tuberosum* ), the world's third most important food crop (FAO 2014), is produced at an annual volume of approximately 383 million metric tons and is widely recognized as a key contributor to global food security (FAO 2019). However, potato production is increasingly threatened by erratic precipitation, temperature extremes, and escalating pest and pathogen pressures (Handayani et al. 2019; Raza and Bebber 2022).

Relocating cultivation to low‐pest regions and cooler latitudes has been proposed as one method to mitigate biotic stress (Cohen et al. 2014; McCrystall et al. 2021; Albrectsen et al. 2025). Yet such relocation alone is insufficient to prevent yield losses, particularly as new and evolving diseases and outbreaks continue to challenge production of potato and other crops. Meanwhile, conventional, chemically intensive agricultural practices are being phased out in response to environmental concerns and regulatory changes. As a result, there is growing interest in restorative and regenerative approaches that can sustainably support crop production (Schreefel et al. 2020). Among these, soil amendments are gaining attention as ecologically sound alternatives to synthetic inputs (e.g., Chen et al. 2021; Biswal et al. 2022; Sun et al. 2023; Henzel et al. 2025; Weiler et al. 2025), that promise to enhance soil health, improve crop performance, and increase resilience to pests and pathogens through direct and indirect mechanisms (FAO 2019; Farrell et al. 2020; Sharma et al. 2025).

In a variety of crops, including potato, straw mulching has been shown to improve yield and productivity under diverse conditions (Ghosh et al. 2006; Kirchner et al. 2014; Wang et al. 2018; Biswal et al. 2022; Khan et al. 2023; Fahad et al. 2024). It has also been associated with reduced herbivory by Colorado Potato Beetles (Weiler et al. 2025) and lower aphid infestation (Kirchner et al. 2014), potentially reducing virus transmission. Returning crop straw to the field is an economically effective way to conserve resources and support sustainable production (Mairata et al. 2023; Sun et al. 2023). Additionally, straw mulch contributes to carbon sequestration, which can help reduce greenhouse gas emissions: by up to 33% in wheat (Genger et al. 2018) and 12.1% in maize (Li, Wang, et al. 2023).

Organic mulches, rich in organic carbon, nitrogen, phosphorus, and other nutrients, may improve soil fertility and support plant growth. By covering the soil surface, straw buffers against moisture loss and temperature extremes, while increasing nutrient availability (Huang et al. 2021; Biswal et al. 2022; Sun et al. 2023). Beyond their physical and nutritional effects, mulches can influence soil microbial community structure and function potentially enriching taxa involved in nutrient cycling and plant health. These microbial communities may also contribute to weed and pest suppression through mechanisms such as competitive exclusion, production of antimicrobial compounds (antibiosis), and activation of plant defenses via induced systemic resistance (e.g., Sharma et al. 2025).

Using ergosterol as a fungal biomarker, Henzel et al. (2025) observed increased abundance of filamentous fungi in mulched potato fields. As an organic substrate, straw promotes the growth of decomposer fungi and bacteria, which break down organic matter and release nutrients accessible to both plants and microbes (Fan and Wu 2020). This release may in turn shift bacterial community composition, favoring r‐strategist, copiotrophic genera that thrive in enriched environments (Li et al. 2021; Shang et al. 2023; Zhang et al. 2024; Yang et al. 2024).

Microbial responses are likely to vary depending on crop, cultivar, and plant compartment (Beckers et al. 2017). Although most studies have focused on the rhizosphere and root‐soil interfaces (İnceoğlu et al. 2010; Weinert et al. 2011; Su et al. 2020; Biswal et al. 2022; Ninkuu et al. 2025), organ‐specific differences suggest that potentially beneficial plant–microbe interactions may occur below ground with direct qualitative impacts, for example, in potato on tuber health.

This study examined the effects of straw mulching on potato‐associated bacterial communities in two cultivars (cv *King Edward* and cv *Mandel*), sampled across four belowground compartments (rhizosphere, root, soil, and tuber peel). Using 16S rRNA amplicon sequencing, we assessed whether mulching altered bacterial abundance and community composition in a source‐ and cultivar‐dependent manner.

## 2 Materials and Methods

### 2.1 Experimental Site

The experiment was conducted from June to September 2023 at the Röbäcksdalen field research station (63°48′32.0″N, 20°13′51.1″ E), Swedish University of Agricultural Sciences, Umeå, Sweden. The average air temperature, relative humidity, and soil temperature during the growing season were 15.4°C, 84%, and 13.6°C, respectively. The soil was classified as silt loam (71% silt, 13% sand, 9% clay) with 3.9% organic matter and a pH of 5.2. Soil chemical properties included: carbon 26 g kg^−1^, nitrogen 2.5 g kg^−1^, phosphorus 28.6 mg 100 g^−1^, potassium 118.7 mg 100 g^−1^, magnesium 13.1 mg 100 g^−1^, calcium 94 mg 100 g^−1^, iron 83 mg 100 g^−1^, and electrical conductivity 1263 μS cm^−1^.

### 2.2 Experimental Design

The experiment was laid out in a split‐plot design with two levels of soil management, soil with and without barley straw mulching (mulch and control). Two potato cultivars, cv *King Edward* and cv *Mandel*, were assigned to the sub‐plots. These cultivars were selected based on their regional relevance. The *Mandel* cultivar is a local landrace of northern Sweden, while cv *King Edward* for decades has become a nationally preferred cultivar throughout Sweden. Each subplot for both soil practices was replicated three times in a randomized block design. The dimensions of each subplot were 4 m × 2 m with a 1 m distance between two adjacent subplots. In each subplot, on June 12th, 2023, ten potato tubers were planted to a depth of 20 cm with a spacing of 0.25 m. The NPK fertilizer ProMagna 11‐5‐18 (YaraMila) was applied at planting, with a total application of 60:27:98 kg ha^−1^ (N:P:K) in the form of nitrate and ammonium, phosphorus oxide, and potassium nitrate, respectively. Three weeks after fertiliziation barley straw was spread over the plots assigned for mulching, and after ten additional days nondestructive measurements of plant growth attributes were conducted at a weekly base, continuing for eight weeks. No irrigation was applied during the experiment.

### 2.3 Growth Parameters

Weekly measurements included recordings of plant height and chlorophyll content, considering the height of the tallest stem. Chlorophyll measurements were conducted using a chlorophyll meter (Opti‐science CCM‐200 plus). Nine measurements were recorded for each plant in a sub‐plot, summing up to eighty‐one measurements per treatment and cultivar.

### 2.4 Sampling and Analysis

Soil samples for conductivity and element analyses were collected twice during the experimental period using a soil sampler probe. Soil properties were analyzed at Agrilab AB, Uppsala, Sweden. Soil pH was measured in a 1:5 (volume fraction soil suspension) as described in SS‐ISO 10390. Electrical conductivity (μS cm^−1^) was measured as suggested in SS‐ISO 11265 using Mettler Toledo Seven Compact Conductivity. Total carbon (C; SS‐ISO 10694) and nitrogen (N; SS‐ISO 13878) contents (g kg^−1^ soil) were determined by dry combustion using an element analyzer (LECO CN928). Nutrient concentrations were analyzed using the Ammonium Lactate/Acetic Acid Method and measured with a Spectro Blue ICP.

### 2.5 Tuber Analyses

Tuber quality analyses were conducted on samples pooled by cultivar and treatment: cv *Mandel* and cv *King Edward*, controls and mulch treated, respectively. Five tubers (*n* = 5) were randomly chosen from each pooled lot. Starch content was assessed by the Anthrone method (Sadasivam 1996). Ascorbic acid content was measured spectrophotometrically following the method described by Hewitt and Dickes (1961). Specific gravity was determined by weighing (washed and dried) tubers individually in air and water and using the equation by Muller et al. (2023): Specific gravity = weight in air/(weight in air − weight in water). Dry matter (DM) of tubers was determined, after drying tubers at 60°C until steady weight using the formula by Escuredo et al. (2021): DM (%) = (dry weight/fresh weight) × 100. Following harvest, tuber weight was averaged across all tubers within a subplot. Tuber size distribution into size categories was then determined using net with mesh sizes: 35 mm, 45 mm, 55 mm, and > 55 mm.

### 2.6 Collection and Storage of Environmental Samples

Environmental DNA (eDNA) was extracted from four below‐ground sources: rhizosphere, root, soil, and tuber peel. To avoid contamination, samples were collected with gloves and sterile tools in the field and lab. Soil was collected from 0, 10, and 20 cm depths, mixed, and placed in sterile Falcon tubes. Rhizosphere samples were obtained by brushing root‐adhering soil into sterile Eppendorf tubes after removing loose soil. Roots were rinsed in sterile water before being transferred to tubes. Tuber peel samples were sliced with a sterile scalpel. Samples were flash‐frozen on dry ice in the laboratory of the field station and transported to the Umeå Plant Science Centre for storage at −80°C until analysis. For each treatment and cultivar, seven replicates were collected for rhizosphere, root, and soil, and two replicates for tuber peel, totaling 92 samples.

### 2.7 eDNA Extraction, PCR Amplification, Library Preparation and Sequencing

eDNA was extracted from 250 mg of sample using the DNeasy PowerLyzer PowerSoil Kit (QIAGEN, Germany) according to the manufacturer's instructions. Purity and concentration of the DNA extracts were assessed using a Qubit 2.0 Fluorometer (Life Technologies Invitrogen, USA). DNA was diluted to a concentration of 5 ng μL^−1^, and 5 μL was used as a template for PCR amplification. The PCR reaction mix consisted of 12.5 μL of KAPA HiFi HotStart ReadyMix, KK2601 (Roche sequencing store), 10 μM of each primer, 0.02 μg μL^−1^ of bovine serum albumin (BSA), and PCR‐grade water to a final volume of 25 μL. The 16S rRNA V3–V4 hypervariable region of bacteria was amplified using the 338F (5′‐ACTCCTACGGGAGGCAGCA‐3′) and 806R (5′‐GGACTACHVGGGTWTCTAAT‐3′) (Li et al. 2022) primers, overhang with the index sequences (5′‐ACACTCTTTCCCTACACGACGCTCTTCCGATCT‐338F and 5′‐GTGACTGGAGTTCAGACGTGTGCTCTTCCGATCT‐806R) to allow for dual indexing of the samples. PCR amplifications were performed under the following conditions: initial activation at 95°C for 3 min, followed by 25 cycles consisting of denaturation for 30s at 95°C, annealing for 30s at 55°C, extension at 72°C for 30s, followed by a final extension at 72°C for 5 min (Chica et al. 2019). All diluted DNA samples were amplified in triplicate. Triplicate reactions were pooled and quantified with a Qubit 2.0 Fluorometer (Life Technologies Invitrogen, USA). After confirming the successful attachment of sample‐specific index primers, the library was subjected to Illumina NextSeq 2000 (2 × 300 bp) paired‐end (PE) sequencing at NGI SciLife Lab, Stockholm, Sweden.

### 2.8 Bioinformatics Analysis

The bioinformatics analysis pipeline nfcore/ampliseq version 2.10.0 (doi: 10.5281/zenodo.1493841) (Straub et al. 2020), the nf‐core collection of workflows (Ewels et al. 2020), utilizing reproducible software environments from the Bioconda (Grüning et al. 2018) and Biocontainers (da Veiga Leprevost et al. 2017) projects, were used for data processing. Raw Illumina NextSeq 2000 (2 × 300 bp) paired‐end reads were demultiplexed by SciLife Lab, Stockholm, Sweden and delivered as sample‐specific fastq files. The sequence quality was checked using FastQC, and the resulting data were summarized with MultiQC (Ewels et al. 2016). Bacterial primers were trimmed using cutadapt (cutadapt_min_overlap 3) (Martin 2011) and untrimmed sequences were discarded. Sequences that did not contain primer sequences were considered artifacts. Less than 13.1% of the sequences were discarded per sample, and a mean of 96.4% of the sequences per sample passed the filtering. Adapter and primer‐free sequences were processed sample‐wise (independent) with DADA2 (Callahan et al. 2016). DADA2 extracts exact amplicon sequence variants (ASVs) from amplicon data by removing PhiX contamination, trimming reads (trunc_qmin 25), discarding reads with more than two expected errors, reducing sequence errors and dereplicating sequences through quality filtering, denoising, read pair merging and PCR chimaera removal. The obtained ASVs were identified and filtered using Barnap tool (Seemann 2013) to retain only bacterial ASVs by removing those associated with mitochondria, chloroplasts, archaea, and eukaryotes. In the end, 46,470 ASVs were identified as bacteria and used for downstream analyses. Taxonomic classification of the bacterial ASVs was performed using DADA2 and QIIME2 (Bolyen et al. 2019) against the Silva 138.1 prokaryotic SSU database (Quast et al. 2012). The bioinformatic pipeline and analysis were run on the HPC2N server (https://www.hpc2n.umu.se/) at Umeå University.

### 2.9 Metabarcoding Analysis for Bacterial Diversity

The abundance table, generated after the removal of irrelevant taxa (as described above), based on computed ASVs and taxonomic classification, was used to measure bacterial abundance in a sample. The abundance table was processed using the vegan and phyloseq packages in R (version 4.3.3; McMurdie and Holmes 2013; Oksanen et al. 2013). The abundance of bacterial genera across eDNA sources and potato cultivars, was analyzed using the “Venn diagram” and “grid” packages in R (version 4.3.3).

The abundance of ASVs was used as a metric of alpha diversity. The abundance of ASV counts was converted to a matrix using the “as. matrix” function in R, which was subsequently used to calculate alpha diversity with the “hill_taxa” function from the hillR package in R (version 4.3.3; Li 2018). Alpha diversity (i.e., Species Richness and Shannon Diversity indices) was assessed across each eDNA source, treatment, and cultivar. We used the aov and pairwise.t.test functions in R (version 4.3.3) to assess differences in alpha diversity. A three‐way ANOVA was performed using the model: Diversity~source + treatment + cultivar to evaluate the main effects of sample source, treatment, and cultivar. To test for interactive effects between treatment and cultivar, we applied a two‐way ANOVA with the model: Diversity~treatment × cultivar. Finally, pairwise comparisons of diversity indices between treatments were conducted using pairwise *t*‐tests, based on the model: Diversity~treatment.

Beta diversity was assessed using the Bray–Curtis dissimilarity index, calculated from ASV abundance data. Community differences were visualized with non‐metric multidimensional scaling (NMDS) using the metaMDS function in the vegan package in R (version 4.3.3). Shifts in community composition were tested with permutational multivariate ANOVA (PERMANOVA) using the adonis2 function in the vegan package (number of permutations = 999).

The feature table was processed using the “rarefy”, “aggregate” and “parLapply” functions from the Vegan R package to calculate rarefaction curves from bacterial ASVs (46,470 ASVs), which were subsequently plotted using the ggplot2 R package.

### 2.10 Statistical Analysis

Plant growth parameters, tuber quality, yield and size, were tested for normality using the Shapiro–Wilk test. Normally distributed data were analyzed with ANOVA, while non‐normally distributed data were analyzed using the Aligned Rank Transform (ART) in ARTool. ASV counts were compared with paired *t*‐test, and soil properties were analyzed with one‐way ANOVA. All statistical analysis and data plotting were conducted in R (version 4.3.3) using ARTool, vegan, and ggplot2. Packages used for community and diversity analysis are stated above. The data that support the findings of this study are openly available in the “github” repository at DOI: 10.5281/zenodo.16948011, reference BAPoPro.zip.

## 3 Results

### 3.1 Effects of Straw Mulching on Soil and Growth Parameters

**FIGURE 1:**
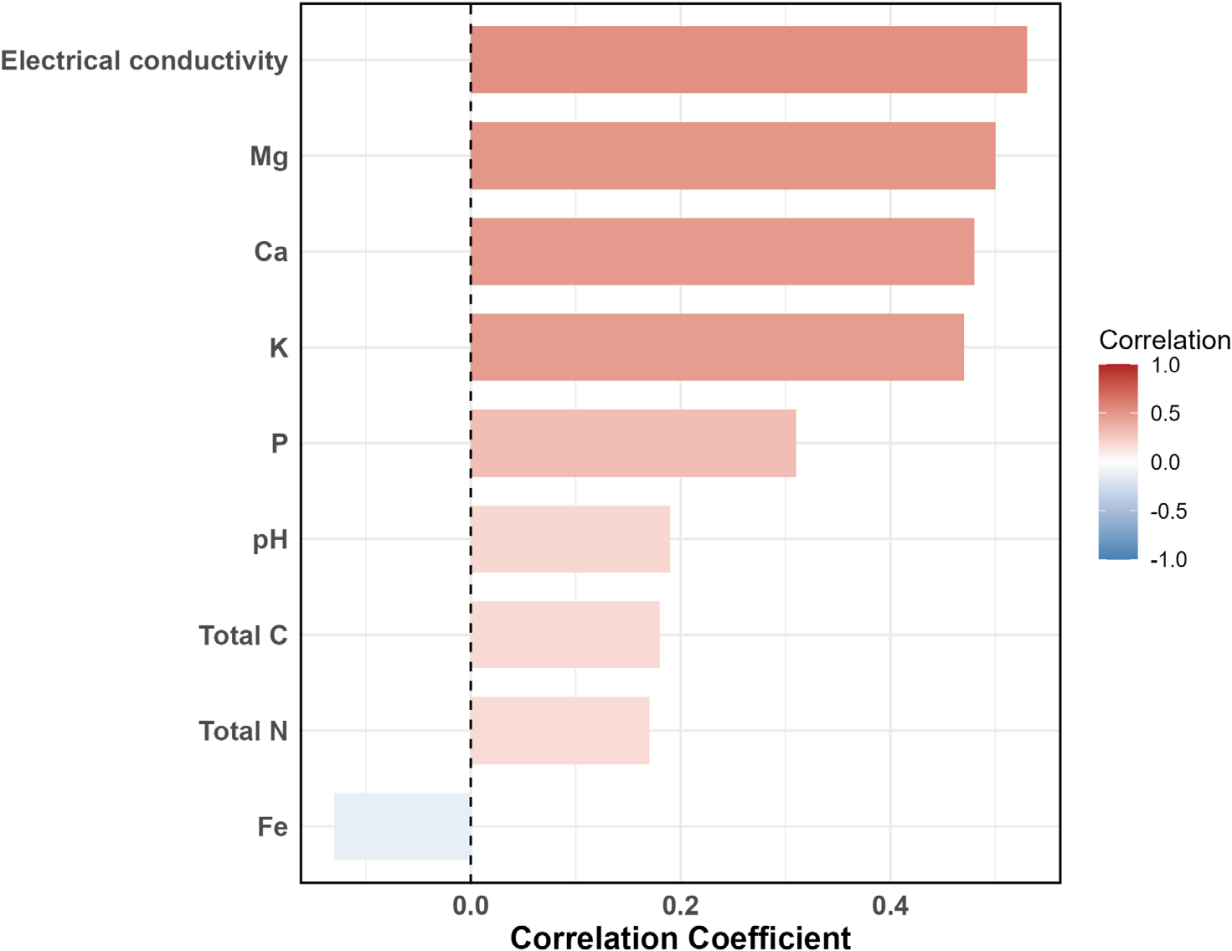
Correlation coefficients between straw mulch treatment and soil physicochemical parameters at harvest. Parameters include elemental concentrations (mg 100 g^−1^ soil), total carbon and nitrogen (g kg^−1^ soil), and electrical conductivity (μS cm^−1^). Bars represent Pearson correlation coefficients, with color indicating direction and strength of the correlation (red = positive; blue = negative).

**FIGURE 2:**
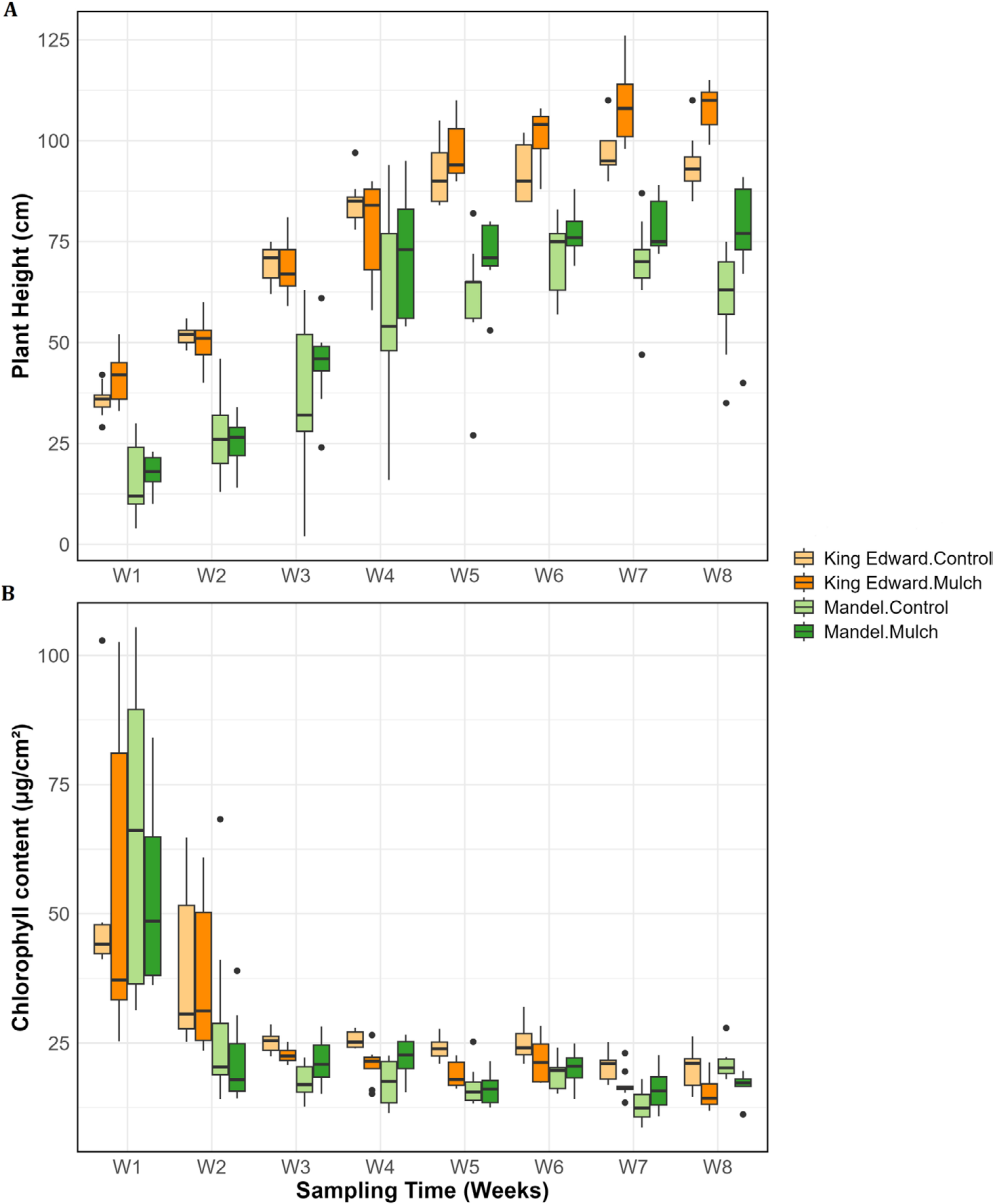
Effects of straw mulching on plant growth traits across the growing season: (A) plant height (cm) and (B) chlorophyll content (μg cm^−2^). Measurements were taken at weekly intervals throughout the season for two potato cultivars: Cv *King Edward* and cv *Mandel*.

As expected, straw mulching tended to increase electrical conductivity (47.8%), phosphorus (11.2%), potassium (40.8%), magnesium (28.6%), and calcium (16.2%) in the mulch‐treated soil (Table S1). Correlations are shown in Figure 1. Plant height increased throughout the experimental period, and this effect was further enhanced by straw mulching. By week 8, plant height in mulched treatments was 14% higher in cv. *King Edward* and 25% higher in cv. *Mandel* compared to controls (*p* < 0.05; Figure 2A; Table 1). Similarly, chlorophyll content was consistently lowered by the mulch treatment across time with cv. *King Edward* having higher contents compared to cv. *Mandel* (Figure 2B; Table 1).

**TABLE 1:** Summary of an aligned rank transform (ART) analysis of variance (ANOVA) model testing the effects of straw mulching (Tr), potato cultivar (cv), time (*T*), and their two‐way interactions nondestructively on plant height and chlorophyll content across the growing season.

	Plant height			Chlorophyll		
	df_1_, df_2_	*F*	*p*	df_1_, df_2_	*F*	*p*
Treatment	1, 254	43.87	2.1e‐10***	1, 238	11.35	8.8e‐4***
Cultivar	1, 254	505.45	< 2.2e‐16***	1, 238	43.21	3.1e‐10***
Time	7, 254	108.27	< 2.2e‐16***	7, 238	37.07	< 2.2e‐16***
Tr × cv	1, 254	3.79	0.05	1, 238	0.77	0.38
Tr × *T*	7, 254	2.28	0.03**	7, 238	1.27	0.27
cv × *T*	7, 254	1.75	0.01	7, 238	2.69	0.01*

*Note:* Significance levels: ***p < 0.001; *p < 0.05; *p* < 0.1.

### 3.2 Effects on Tuber Quality, Yield, and Size

**FIGURE 3:**
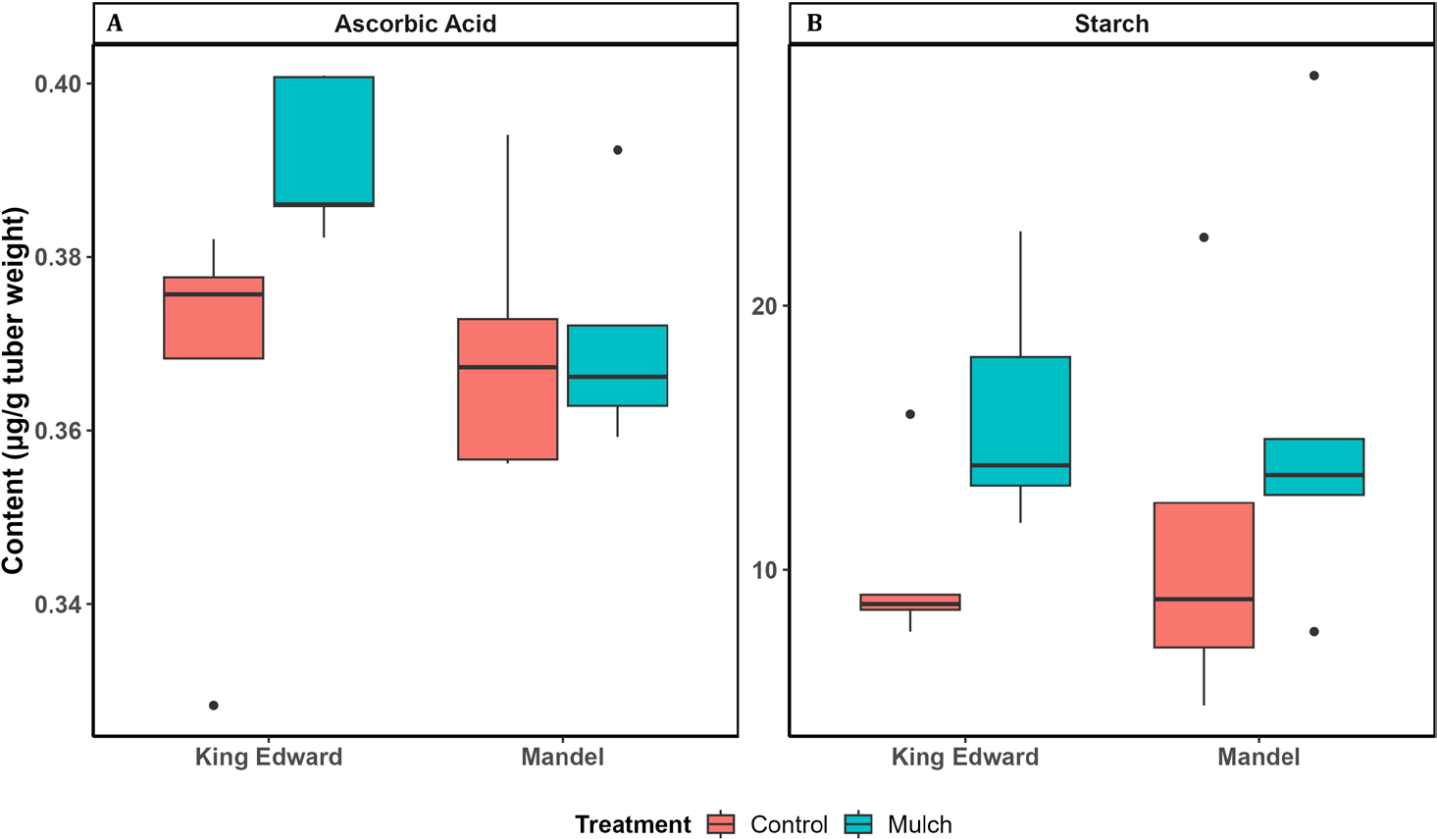
Tuber quality measures: (A) ascorbic acid and (B) starch content (μg g^−1^ fresh tuber weight) in potato cv *King Edward* and cv *Mandel*, grown under two soil conditions: Control (red) and straw mulch (blue).

Effects of mulching, cultivar, and their interaction on tuber quality and quantity parameters are summarized in Table 2. The results indicate a strong cultivar effect on tuber dry matter content and yield. Treatment effects were most strongly seen for tuber starch that increased 28%–37% in response to mulch (Table S2; *F* = 3.79, *p* = 0.007; *n* = 5 per treatment and cultivar) (Figure 3; Table S2). Ascorbic acid concentrations increased in cv *King Edward* from 0.37–0.39 mg g^−1^ (*p* < 0.05; *n* = 5 per treatment). Tuber size class distribution varied between cultivars and in cv *Mandel*, straw mulching resulted in a four‐fold increase in the proportion of tubers within the second‐largest size class (55 mm) (Table S2).

**TABLE 2:** Summary of analysis of variance (ANOVA) testing the effects of straw mulching treatment (Tr), potato cultivar (C), and their interaction (Tr × C) on the following traits: Starch content *in μg g* ^*−1*^ *tuber*, ascorbic acid content *in μg g* ^*−1*^ *tuber*, dry matter *in %*, specific gravity, and tuber yield *in g plant* ^*−1*^.

Response	Treatment		Cultivar		Tr × C	
	*F*_(df = 1)_	*p*	*F*_(df = 1)_	*p*	*F*_(df = 1)_	*p*
Starch	3.79	0.07	0.02	0.88	0.09	0.76
Ascorbic acid	3.46	0.08	1.60	0.22.	2.87	0.11
Dry matter	0.24	0.63.	9.46	0.007**	0.50	0.49
Specific gravity	0.94	0.35.	0.78	0.39	1.20	0.29
Tuber yield	0.08	0.79	25.92	0.001***	1.06	0.33

*Note:* Model residuals were assessed for normality using the Shapiro–Wilk test. Significance levels: ***p < 0.001; **p < 0.01; *p* < 0.1.

### 3.3 Effects on Bacterial Richness and Alpha Diversity

**FIGURE 4:**
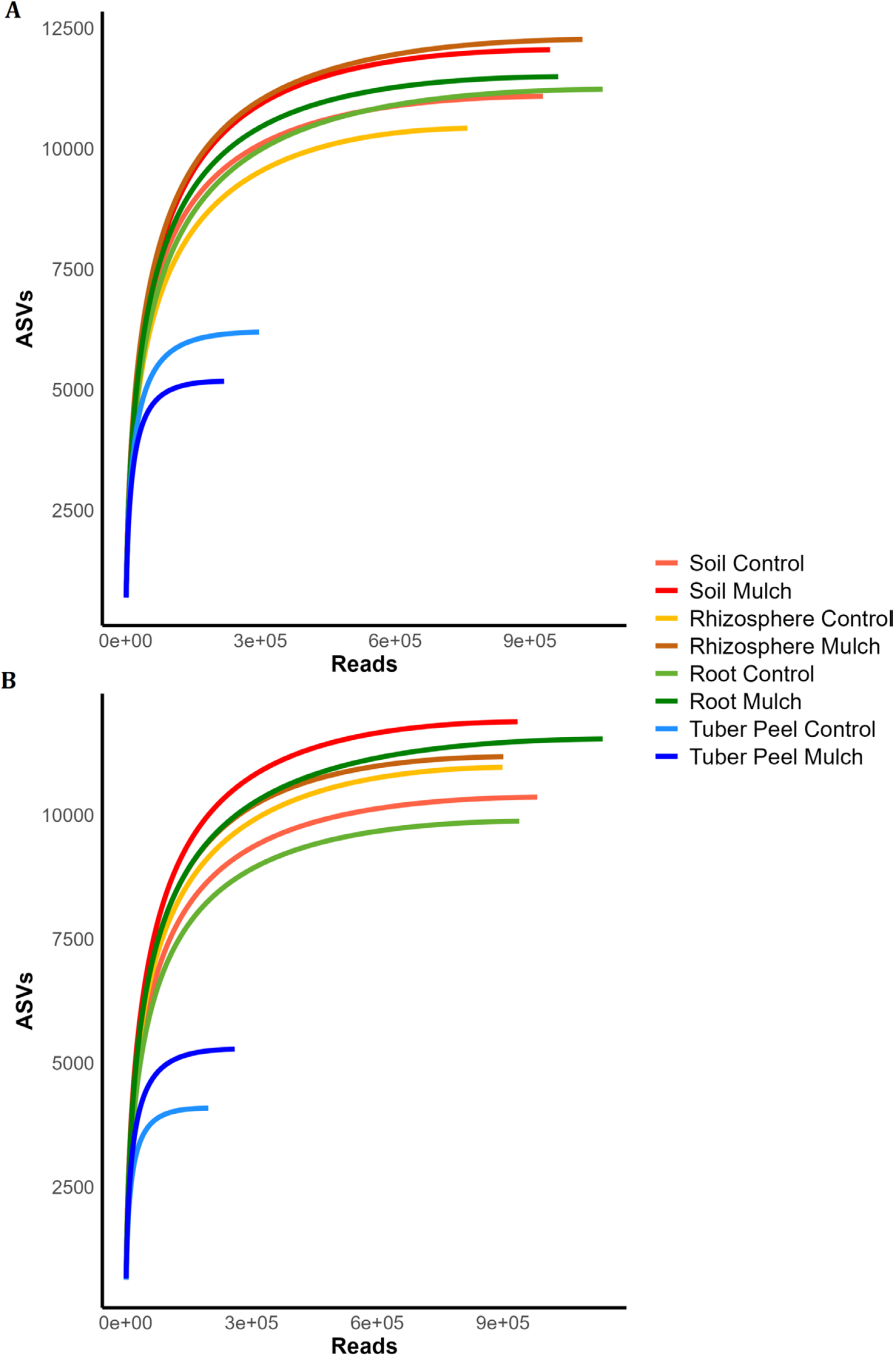
Rarefaction curves based on amplicon sequence variant (ASV) counts, illustrating the effect of straw mulching on bacterial richness in two potato cultivars: (A) cv *King Edward* and (B) cv *Mandel*. Sample sources include rhizosphere, root, soil, and tuber peel. Color coding corresponds to sample type and treatment, as shown in the legend.

A total of 23,286,029 raw sequencing reads were obtained from 92 potato field samples representing four eDNA sources: rhizosphere, root, soil, and tuber peel. After quality and ASV filtering, 12,452,537 reads remained, corresponding to 46,470 ASVs, with an average of 135,353 reads per sample (range: 82,856–216,116 reads). Rarefaction curves based on the filtered reads indicated bacterial species richness adjusted according to sampling effort (Figure 4A,B). Overall, straw mulching increased bacterial richness in both cultivars across all sample types. Additionally, ASV abundance was lowest in tuber peels.

### 3.4 Effects on Bacterial Community Composition

**FIGURE 5:**
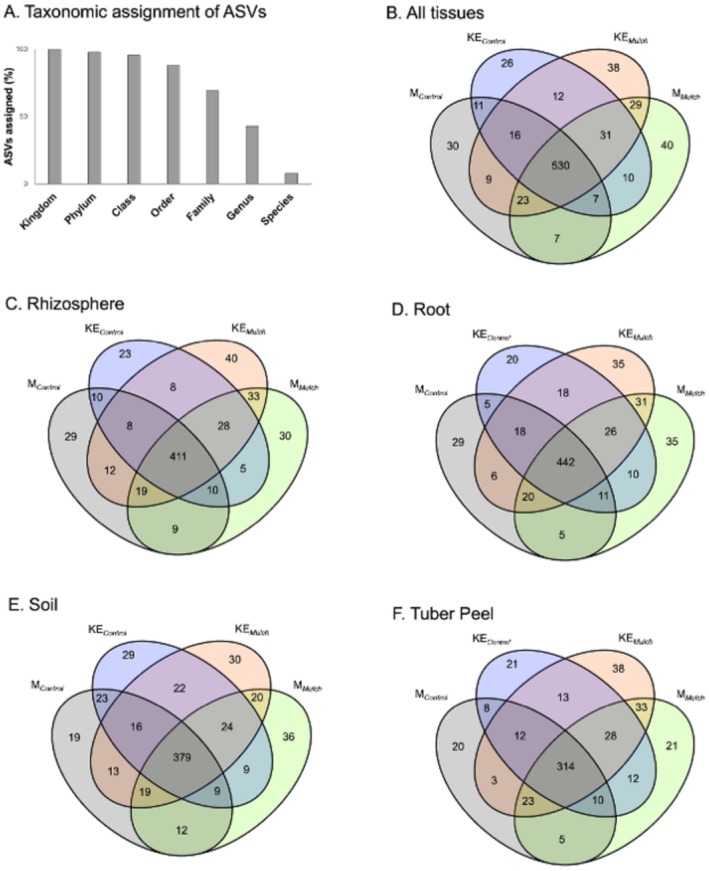
Taxonomic resolution of bacterial communities in potato, representing two cultivars, four sample types, and two treatments, based on 16S ribosomal RNA (rRNA) gene sequencing (Illumina platform). Amplicon sequence variants (ASVs) were inferred using DADA2, a denoising algorithm that models and corrects sequencing errors in Illumina amplicon data, and processed through the nf‐core/ampliseq pipeline (version 2.10.0) using the SILVA 138.1 small subunit (SSU) database for taxonomic assignment. (A) Taxonomic classification of ASVs across all samples. (B) Venn diagram of bacterial genera across all samples. (C–F) Venn diagrams divided after sample type: (C) rhizosphere, (D) root, (E) soil, and (F) tuber peel. Each diagram presents unique and shared genera by treatment (mulch, M; control, C) and cultivar (cv *King Edward*, KE; cv *Mandel*, M).

Taxonomic classification of the bacterial ASVs was performed using DADA2 against the SILVA 138.1 prokaryotic SSU database, which identified only 7.93% at the species level and 43.15% at the genera level (Figure 5A). Therefore, genus‐level classification was used for the taxonomic characterization of the bacterial communities. Venn diagrams display the effects of straw mulching and cultivar on shared and unique bacterial genera across all eDNA sources (Figure 5B) and for each eDNA source alone (Figure 5C).

A total of 530 ASVs were shared between the two cultivars under both treatments. Compared to control conditions, the number of unique ASVs was notably higher under mulch (cv *King Edward* = 46%, cv *Mandel* = 33%). The increase in ASVs specific to straw mulching across the four eDNA sources was also statistically significant (*p* < 0.01) and more pronounced in cv *King Edward* (*p* < 0.01) compared to cv *Mandel* (*p* > 0.1), with increases of 74%, 75%, 3.5%, and 81% for the rhizosphere, root, soil, and tuber peel, respectively.

**FIGURE 6:**
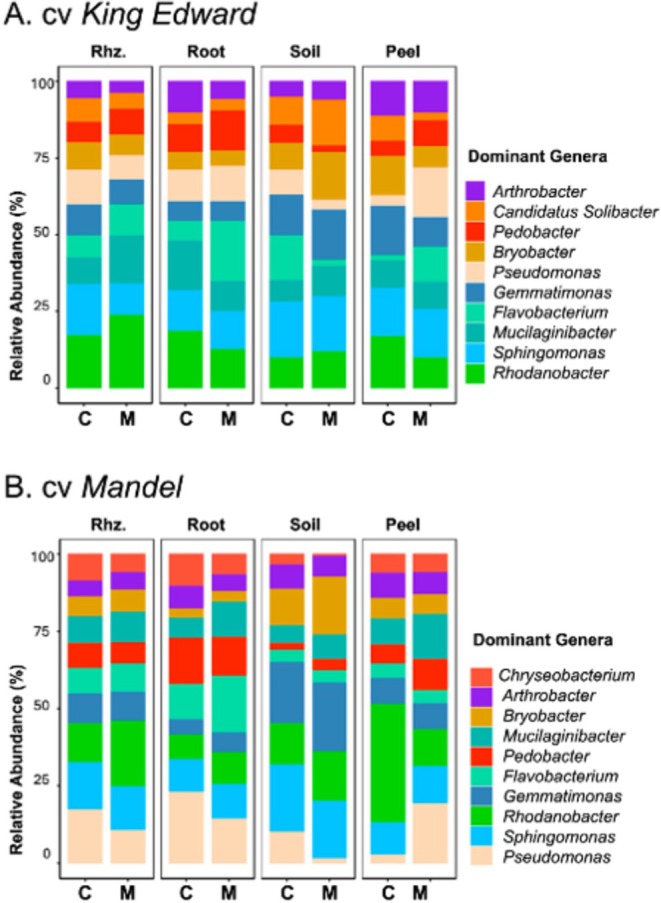
Relative Abundance (percent) of the top 10 bacterial genera in two potato cultivars: cv King Edward (A) and cv Mandel (B). Within each cultivar, data are shown by sample type equivalent to environmental DNA source from: Rhz. (Rhizosphere), Root, Soil, and Peel (tuber peel) and by treatment: C (control) and M (straw mulch‐enriched soil). Genera are color‐coded consistentently across cultivars and stacked in order of decreasing relative abundance within a cultivar (most abundant at the base).

To compare the taxonomic distribution across source and cultivar, the 10 most abundant genera were displayed (Figure 6), excluding unassigned taxa (accounting for approximately 65% of total community abundance). Mulching generally increased the relative abundance of genera associated with plant growth promotion and organic matter decomposition.

In the cv *King Edward* rhizosphere, mulching increased the relative abundance of *Mucilaginibacter* (1.8 fold), followed by *Rhodanobacter* and *Flavobacterium* (1.4 fold each). In the root, *Flavobacterium* and *Pedobacter* increased 3‐ and 1.5‐fold, respectively. In the soil, *Bryobacter* and *Candidatus Solibacter* increased by 1.8‐ and 1.6‐fold. In the tuber peel, *Pseudomonas*, *Flavobacterium*, and *Pedobacter* increased by 5‐, 5.8‐, and 1.7‐fold, respectively, while those of *Bryobacter* and *Candidatus Solibacter* decreased (Figure 6A). A similar pattern was observed in cv *Mandel*, where the relative abundances increased of *Rhodanobacter* in the rhizosphere; *Flavobacterium* and *Mucilaginibacter* in the root; *Bryobacter* in the soil; and *Pseudomonas*, *Mucilaginibacter*, and *Pedobacter* in the tuber peel, with fold changes ranging from 1.6 to 7 (Figure 6B).

These shifts in genus‐level abundance across eDNA sources, which were visible in both cultivars, highlight the potential of plant compartment in shaping bacterial community composition in response to mulching.

### 3.5 Dissimilarities Among Bacterial Communities

**FIGURE 7:**
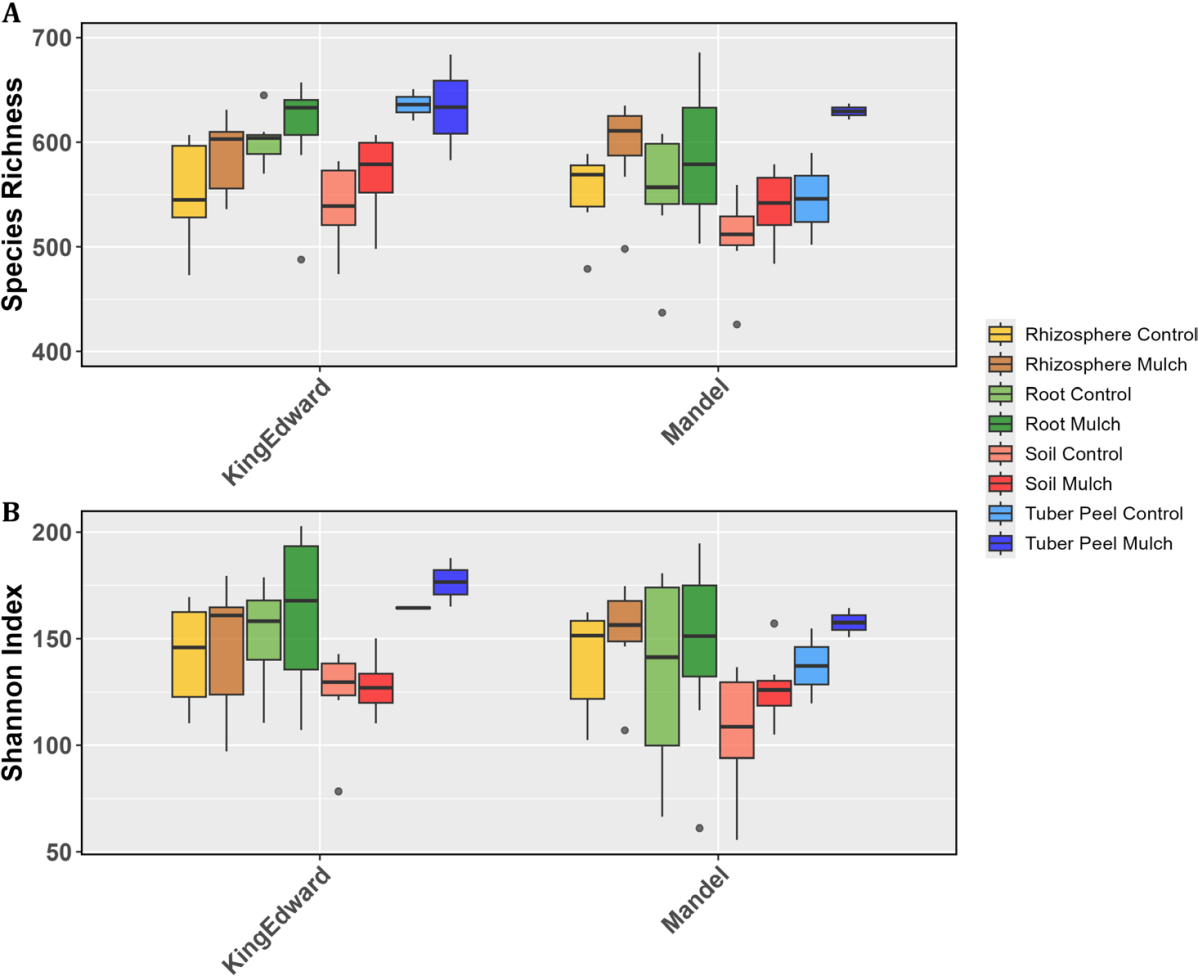
Community structure indicators: (A) species richness and (B) Shannon diversity index of below‐ground bacterial communities associated with potato (cv *King Edward* and cv *Mandel*). Boxplots present the median (central line), with the box edges indicating the first (Q1) and third (Q3) quartiles. Points beyond the whiskers represent potential outliers. Color coding in legend details sample source (rhizosphere, root, soil, and tuber peel) and treatment (control and straw mulch).

Alpha diversity indices (Species Richness and Shannon) were assessed based on ASVs across four eDNA sources, two treatments, and two cultivars, and reflected variation in bacterial community richness (Figure 7a) and evenness (Figure 7b), respectively. eDNA sources had strong effects on both alpha diversity indices (*p* < 0.0001; Table 3). Moreover, both potato cultivars followed similar overall patterns, with increased diversity in response to mulching. However, the mulching treatment effect was generally weaker for species richness (*p* < 0.02) than for evenness (*p* < 0.07). Additionally, plant‐associated samples appeared to exhibit higher alpha diversity values compared to soil samples, with the strongest treatment‐related increase observed in tuber peel samples of cv. *Mandel*.

**TABLE 3:** Summary of analysis of variance (ANOVA) testing the effects of environmental sample source, straw mulching treatment, and potato cultivar on two alpha diversity indices: Species richness and Shannon diversity of bacterial communities associated with field‐grown potato.

Effect	Species richness					Shannon index				
	df	SS	MS	*F*	*p*	df	SS	MS	*F*	*p*
Source	3	49,370	16,457	8.04	8.7 e‐05***	3	15,109	5036	6.49	5.2 e‐4***
Treatment	1	21,640	21,640	10.58	0.002**	1	2632	2632	3.39	0.07.
Cultivar	1	11,870	11,870	5.80	0.02*	1	2031	2031	2.62	0.11

*Note:* Significance levels: ***p < 0.001; **p < 0.01; *p < 0.05; *p* < 0.1.

**FIGURE 8:**
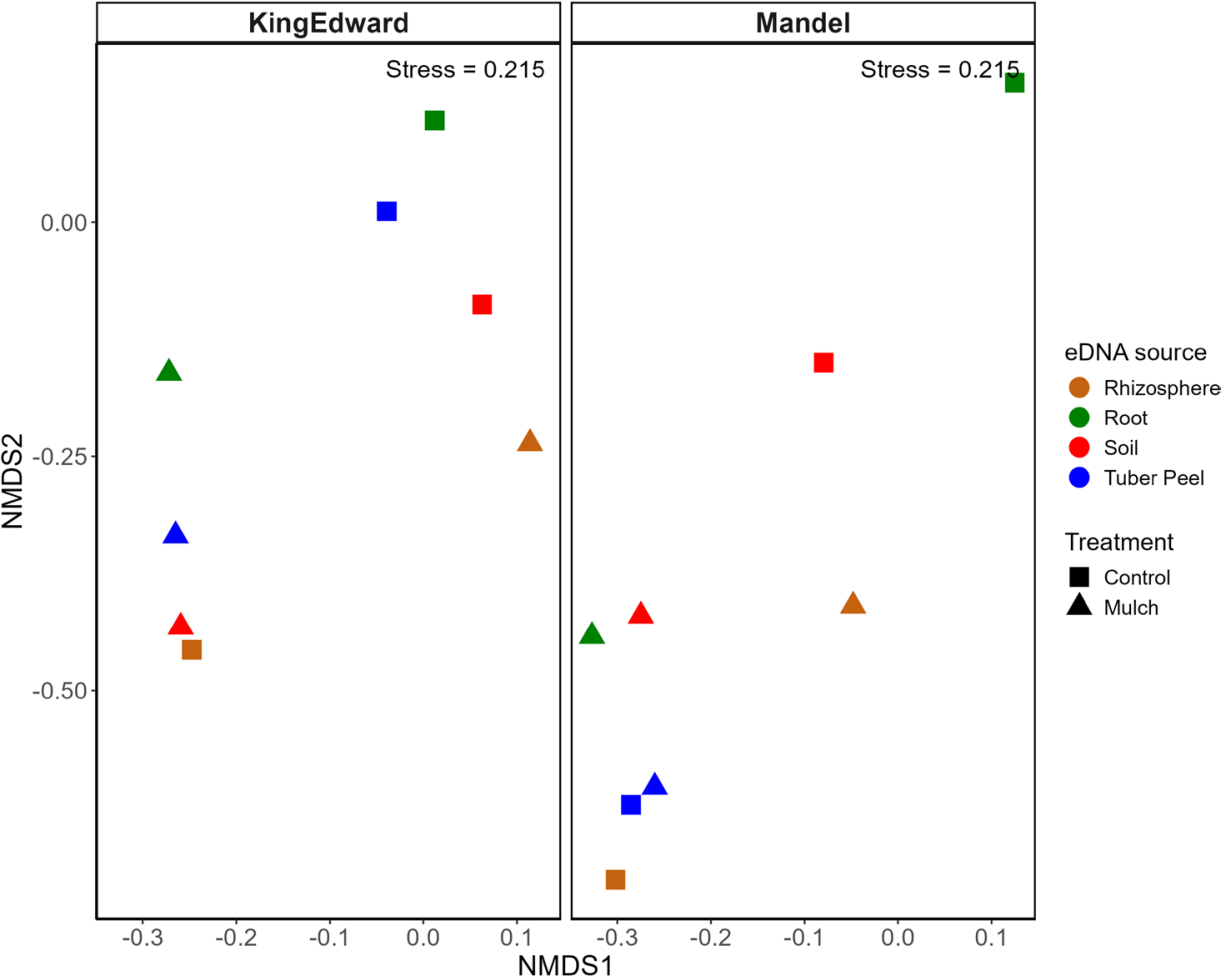
Non‐metric multidimensional scaling (NMDS) plots based on Bray–Curtis dissimilarities, showing the effect of straw mulching on bacterial community structure across eDNA sample sources in two potato cultivars (cv *King Edward* and cv *Mandel*). “Stress” indicates the goodness‐of‐fit of the NMDS ordination. Closer proximity of points indicates greater similarity in community composition (lower beta diversity), while more widely dispersed points reflect greater dissimilarity (higher beta diversity). eDNA, environmental DNA; treatment, mulch treatment using barley straw.

Non‐metric multidimensional scaling (NMDS) is an ordination method that visualizes similarity among samples. For our experiment, mulch‐treated samples appeared to cluster more tightly, compared to control samples, with similar patterns observed for both cv. *King Edward* and cv. *Mandel* (Figure 8). Although NMDS interpretations should be treated with caution when “Stress” values exceed 0.2 (Chase and Myers 2011; Legendre and De Cáceres 2013), the ordination patterns were consistent with the alpha diversity results.

Mulched samples exhibited lower multivariate dispersion, indicating reduced beta diversity and greater community homogeneity across replicates. In contrast, control samples showed lower within‐sample diversity (Figure 7) but higher beta diversity, reflecting greater variability in community composition (Figure 8).

These patterns were further supported by PERMANOVA analyses, which revealed strong effects of eDNA source (*p* = 0.001), as well as interactive effects between treatment and cultivar (*p* = 0.001) and among source, treatment, and cultivar (*p* = 0.001). In contrast, the PERMANOVA did not suggest single effects of treatment or cultivar (*p* > 0.05; Table 4).

**TABLE 4:** Model summary of permutational multivariate analysis of variance (PERMANOVA) testing the effects of sample source (Ss), straw mulching treatment (Tr), potato cultivar (cv), and their two‐ and three‐way interactions bacterial communities associated with field‐grown potato. Community dissimilarities were calculated using Bray–Curtis distances.

Effect	df	SS	*R* ^2^	*F*	*p*
Sample source	3	1.88	0.22	8.38	0.001***
Treatment	1	0.11	0.01	1.23	0.24
Cultivar	1	0.14	0.02	1.49	0.14
Ss **×** Tr	7	2.21	0.26	4.26	0.001***
Ss **×** cv	7	2.22	0.26	4.28	0.001***
Tr **×** cv	3	0.31	0.04	1.11	0.31
Ss **×** Tr **×** cv	15	2.81	0.33	2.53	0.001***

*Note:* Significance levels: ***p < 0.001.

Together, these results highlight a potential decoupling of alpha and beta diversity, reflecting differences in community assembly processes or environmental filtering mainly across sample sources and treatment.

## 4 Discussion

This study investigated how barley straw mulching influences the composition and diversity of potato‐associated bacterial communities across two potato cultivars and four belowground compartments. Using 16S rRNA amplicon sequencing, we found that bacterial community composition was primarily shaped by eDNA source, with additional cultivar‐ and treatment‐dependent effects.

Mulching significantly increased bacterial richness, with the strongest increases observed in the rhizosphere that promoted genera associated with organic decomposition and plant growth promotion. Mulching further enhanced starch and ascorbic acid concentrations in cv *King Edward* tubers and shifted tuber size distribution in cv *Mandel*, suggesting cultivar‐specific benefits. These findings highlight the potential of straw mulching to modulate plant–microbe interactions, and emphasize that plant compartment and cultivar may shape responses differently.

Straw mulching has been proposed as a sustainable strategy to reduce pesticide reliance and mitigate climate‐related stress in potato cultivation (Sun et al. 2023). In addition to influencing nutrient availability and microbial communities (Cong et al. 2020; Waheed et al. 2023; Ninkuu et al. 2025), mulching may also suppress weeds, pests, and diseases (Kirchner et al. 2014; Huang et al. 2021; Waheed et al. 2023), it may also. In our study, plant height increased under mulch (Figure 2; Table S1), consistent with findings in maize (Khan et al. 2022), sunflower (Zhao et al. 2014), and potato (Waheed et al. 2023). In contrast, chlorophyll content did not vary during the study period, which is in line with time‐ and cultivar‐dependent responses reported in fenugreek and peanut (Raheem Lahmod et al. 2019; Ghosh et al. 2006).

Soil nutrient availability (particularly nitrogen, potassium, and Phosphorous) is a key determinant of potato yield and quality (Agbede 2010; Jasim et al. 2020). In this study, nutrient increases under mulch were modest. Nonetheless, changes in microbial diversity likely contributed to improved tuber traits. We observed increased starch and ascorbic acid content in *King Edward* tubers under mulch (Table S2; Figure 3) and althought overall yields were not strongly affected in our study, mulching altered tuber size distribution in *Mandel* towards average larger sizes in agrement with earlier reports (Zahed et al. 2022; Zhang et al. 2023).

Straw mulching increased bacterial richness across cultivars and belowground compartments, in form of elevated ASV counts (Figure 4). However, the Shannon diversity index did not increase, which could be due to uneven distribution of taxa. While the Spices Richness Index captures rare taxa, the Shannon Index accounts for both richness and evenness, and mulching may favor low‐abundance taxa without substantially altering the dominance structure (Jiao et al. 2018, 2019). Additionally, the evenness may be impacted by the application method and experimental duration (Tang et al. 2021), at times even suppressing diversity (Zhang et al. 2024).

While bacterial reference databases are generally more developed and standardized than those for other groups of microorganisms, such as filamentous fungi (Djemiel et al. 2017; Nilsson et al. 2019; Tedersoo et al. 2022), species‐level taxonomic resolution remains constrained by the availability and quality of reference genomes and molecular identifiers (Keck et al. 2023; Schrader et al. 2025). Of ASVs in our study only 7.93% were classified to species level using SILVA 138.1, and approximately 65% of reads remained unassigned at lower taxonomic levels. Nonetheless, genus‐level analyses revealed clear shifts in community structure under mulching (Figs. 5 and 6). Mulching enriched genera associated with plant‐beneficial traits, including *Rhodanobacter*, *Mucilaginibacter*, and *Flavobacterium* in the rhizosphere and root, and *Bryobac*ter, *Candidatus Solibacter*, and *Pseudomonas* in soil and tuber peel. Interestingly, we found a repeated enrichment of *Pseudomonas* in tuber peel, which might be attracted through particular exudates from the peel (Chen et al. 2022). The genus, *Pseudomonas*, is known for its versatility and plant‐beneficial functions (Panpatte et al. 2016; Sharma et al. 2025), and this finding together with enhanced starch and ascorbic acid contents in the tubers could indicate a mutual and positive feedback mediated by the mulch treatment.

Shifts in abundances suggest functional transitions within the microbial communities. Copiotrophic taxa such as *Proteobacteria and Bacteroidota* were favored under mulching, while oligotrophic *Acidobacteriota* declined. This aligns with resource preferences of these groups (Li et al. 2021; Li, Zhu, et al. 2023; Shang et al. 2023; Khomutovska et al. 2024; Yang et al. 2025). Straw application may create nutrient‐rich niches that support fast‐growing r‐strategists capable of degrading complex organic matter, as also reported in mulched maize rhizospheres (Yang et al. 2024). Environmental buffering may promote aerobic mesophiles like *Rhodanobacter*, known for nitrogen cycling and metal tolerance (Peng et al. 2022) and plant growth promoting potentials (Woo et al. 2024). Complex polysaccharides from straw provide substrates for genera like *Flavobacterium* and *Mucilaginibacter*, which degrade plant residues besides producing antimicrobial compounds (Khomutovska et al. 2024; Yang et al. 2025).

In our study, straw mulch altered microbial composition across plant compartments, with NMDS analysis confirming separation by eDNA source (Figure 7). This supports earlier findings of compartment‐specific microbial communities as reported for *Populus* (Gottel et al. 2011; Beckers et al. 2017), *Arabidopsis* (Lundberg et al. 2012; Bulgarelli et al. 2012), potato (Chen et al. 2022; Martins et al. 2024), and rice (Edwards et al. 2015).

Organic mulching remains relatively underexplored in modern agricultural systems (Schreefel et al. 2020). However, recent studies have begun to investigate how mulch composition, application rate, and frequency influence microbial evenness and richness. These studies suggest that providing diverse and sustained carbon sources can support more complex microbial networks and contribute to improved soil health (Zhang et al. 2020; Liu et al. 2025). Yet, despite growing scientific interest, sustainable practices like organic mulching often remain confined to experimental trials. To move beyond isolated case studies, it is critical to diversify research approaches systematically, for example supported by longer‐term and farm‐scale validations (Zhang et al. 2020; Al‐Shammary et al. 2024) and cross‐border collaborations (Albrectsen et al. 2025).

In this context, future work should not only optimize straw mulching under different production systems but also examine the performance of alternative crop residues—such as wheat, rice, or corn straw—in promoting beneficial microbiomes and improving plant health. Doing so may help identify mulch types best suited to local conditions, while contributing to broader goals of sustainability, resilience, and conscientious agricultural development.

## 5 Conclusion

Our study highlights the multifaceted effects of barley straw mulching on potato cultivation. Mulching increased bacterial richness and favored copiotrophic, plant‐beneficial genera such as *Rhodanobacter*, *Pseudomonas*, *Flavobacterium*, and *Mucilaginibacter*, linked to organic matter degradation and plant growth promotion. Bacterial community structure was most strongly influenced by eDNA source, although treatment and cultivar interactions were evident. The enrichment of *Pseudomonas* in tubers coincided with increased starch and ascorbic acid levels. These findings suggest that straw mulching could enhance tuber quality in potato through microbiome‐mediated effects, potentially by enriching beneficial bacterial taxa in a cultivar‐ and tissue‐specific manner.

## Funding

This work was funded by the Kempe Foundation (Grant No. JCSMK23‐0066) and the Stiftelsen Gunnar och Ruth Björkmans fond för norrländsk botanisk forskning. The Knut and Alice Wallenberg Foundation (Grants no KAW 2016.0352 and KAW 2020.0240) financially supported the infrastructure at the Umeå Plant Science Centre.

## Conflicts of Interest

The authors declare no conflicts of interest.

## Supporting information


**Table S1:** Comparative analysis of soil physicochemical properties at harvest under two conditions: with (Mulch) and without straw mulching (Control). Values represent mean (*n* = 3) ± standard error. The *p*‐values, obtained through one‐way ANOVA (Parameter~Treatment).
**Table S2:** Effect of straw mulching on tuber quality and yield traits for two potato cultivars: cv *King Edward* and cv *Mandel*. The traits include ascorbic acid, dry matter, specific gravity, starch content. Values represented in the table are mean ± SE (*n* = 5). Tuber weight, yield, and size distribution are assessed from the entire harvest. *p* values are calculated based on one‐way ANOVA, indicating the significance of treatment (Control and Mulch) effects within each cultivar; *p < 0.05.

## Data Availability

The data that support the findings of this study are openly available in the “github” repository at DOI: 10.5281/zenodo.16948011, reference BAPoPro.zip.
