# Supplementary material for: Straw Mulching Differentially Shapes the Structure and Function of Below‐Ground Bacterial Communities in Potato Depending on eDNA Source and Cultivar
Source: Plant Environ Interact. 2026 Feb 19;7(1):e70131. doi: 10.1002/pei3.70131 (PMC12921270; doi:10.1002/pei3.70131)
Supplement: Supplementary file 1 — Table S1: Comparative analysis of soil physicochemical properties at harvest under two conditions: with (Mulch) and without straw mulching (Control). Values represent mean (n = 3) ± standard error. The p‐values, obtained through one‐way ANOVA (Parameter~Treatment). Table S2: Effect of straw mulching on tuber quality and yield traits for two potato cultivars: cv King Edward and cv Mandel. The traits include ascorbic acid, dry matter, specific gravity, starch content. Values represented in the table are mean ± SE (n = 5). Tuber weight, yield, and size distribution are assessed from the entire harvest. p values are calculated based on one‐way ANOVA, indicating the significance of treatment (Control and Mulch) effects within each cultivar; *p < 0.05. [file PEI3-7-e70131-s001.docx]

Supplementary Material:

Table S1:

Comparative analysis of soil physicochemical properties at harvest under two conditions: with (Mulch) and without straw mulching (Control). Values represent mean (n = 3) ± standard error. The p-values, obtained through one-way ANOVA (Parameter ~ Treatment).

| **Parameters** | **Control** | **Mulch** | **P** |
| --- | --- | --- | --- |
| Electrical conductivity (µS cm*^-1^*) | 215.13 ± 31.32 | 318.13 ± 76.36 | 0.28 |
| Magnesium (*mg 100 g^- 1^soil*) | 3.5 ± 0.31 | 4.5 ± 0.81 | 0.31 |
| Calcium (*mg 100 g^-1^ soil*) | 80.33 ± 7.22 | 93.33 ± 9.24 | 0.33 |
| Potassium (*mg 100 g^-1^ soil*) | 16.6 ± 2.50 | 23.37 ± 5.85 | 0.35 |
| Phosphorous (*mg 100 g^-1^ soil*) | 11.9 ± 1.36 | 13.23 ± 1.56 | 0.56 |
| pH | 5.1 ± 0.03 | 5.2 ± 0.17 | 0.73 |
| Total Carbon (g Kg*^-1^* soil) | 25.14 ± 1.08 | 25.91 ± 1.76 | 0.73 |
| Total Nitrogen (*g Kg^-1^ soil*) | 1.94 ± 0.07 | 1.99 ± 0.13 | 0.74 |
| Fe (*mg 100 g^-1^  soil*) | 82 ± 3.61 | 81 ± 1.53 | 0.81 |

Table S2:

Effect of straw mulching on tuber quality and yield traits for two potato cultivars: cv *King Edward* and cv *Mandel*. The traits include ascorbic acid, dry matter, specific gravity, starch content. Values represented in the table are mean ± SE (n = 5). Tuber weight, yield, and size distribution are assessed from the entire harvest. P values are calculated based on one-way ANOVA, indicating the significance of treatment (Control and Mulch) effects within each cultivar; * = p < 0.05.

| **Responses** | **King Edward** | | | | **Mandel** | | |
| --- | --- | --- | --- | --- | --- | --- | --- |
|  | **Control** | | **Mulch** | **P** | **Control** | **Mulch** | **P** |
|  | **Mean ± SE** | | **Mean ± SE** |  | **Mean ± SE** | **Mean ± SE** |  |
| Ascorbic acid  (*µg g^-1^  tuber weight*) | 0.37 ± 0.00 | | 0.39 ± 0.00 | 0.05 * | 0.37 ± 0.01 | 0.37 ± 0.01 | 0.90 . |
| Dry matter (%) | 21.45 ± 1.62 | | 23.03 ± 0.64 | 0.39 . | 26.45 ± 1.91 | 26.17 ± 0.58 | 0.90 . |
| Specific gravity | 1.08 ± 0.01 | | 1.09 ± 0.01 | 0.21 . | 1.09 ± 0.01 | 1.09 ± 0.01 | 0.93 . |
| Starch content  (*µg g^-1^  tuber weight*) | 9.97 ± 1.50 | | 15.96 ± 2.01 | 0.04 * | 11.19 ± 3.11 | 15.55 ± 3.51 | 0.38 . |
| Tuber weight (*Kg*) | 5.63 ± 0.35 | | 5.30 ± 0.18 | 0.57 . | 1.34 ± 0.49 | 1.51 ± 0.04 | 0.57 . |
| Tuber yield (*Kg plant^-1^*) | 0.94 ± 0.06 | | 0.88 ± 0.03 | 0.57 . | 0.22 ± 0.08 | 0.25 ± 0.01 | 0.57 . |
| Tuber size distribution | 35 mm | 6.4% | 10.50% | 0.56 . | 45.4% | 39.78% | 0.32 . |
|  | 45 mm | 24.9% | 18.72% | 0.32 . | 47.4% | 39.78% | 0.85 . |
|  | 55 mm | 57.4% | 52.51% | 0.33 . | 5.2% | 20.43% | 0.02 * |
|  | > 55 mm | 11.3% | 18.27% | 0.21 . | 2.1% | 0% | 0.37 . |
